# Hepatic epithelioid hemangioendothelioma with *TFE3* rearrangement: a case report and literature review

**DOI:** 10.3389/fonc.2025.1442233

**Published:** 2025-01-23

**Authors:** Ke Meng, Xingrong Yang, Sitong Guo, Juan Tao

**Affiliations:** Department of Pathology, The Second Affiliated Hospital of Dalian Medical University, Dalian, China

**Keywords:** epithelioid hemangioendothelioma, TFE3 rearrangement, liver, imaging features, histopathologic features, case report

## Abstract

Epithelioid hemangioendothelioma (EHE) is a rare low-grade malignant tumor of vascular origin. It may be confusing as its manifestations of multifocal lesions on imaging and epithelial histomorphology in pathology. EHE is easy to be mistaken for a metastatic tumor by radiologists and clinicians. Correct diagnosis and therapy are important owing to the variable clinical course and special treatment. EHEs harbor major *CAMTA1* rearrangement and <5% *TFE3* rearrangement. Meanwhile, EHE with *TFE3* rearrangement has distinctive morphology features. Currently, there are only two cases of hepatic EHE with *TFE3* rearrangement reported, we present another case here that occurred in a 34-year-old female. Both the clinician and radiologist provisionally considered it as a metastatic tumor. The tumor cells have mild atypia but infiltrative growth patterns like benign vascular tumors. Our case is unique mainly in that the absence of its characteristic well-defined vessels, and the presence of unreported morphology of intraluminal papillary proliferation of tombstone or hobnail endothelial cells. The final diagnosis of EHE with *TFE3* rearrangement was made by combining morphological, immunohistochemical, and molecular test results. The patient did not receive any treatment according to her condition and no change was detected in the mass’s size and number on CT images during 3.5 years of follow-up.

## 1 Introduction

Epithelioid hemangioendothelioma (EHE) is a rare malignant vascular tumor, that often occurs in soft tissues/bones or visceral sites, such as the liver and lung. Histologically, it is composed of epithelioid endothelial cells arranged in cords, nests, or single cells in the myxohyaline stroma. Tumor cells don’t have strong angiogenic properties commonly. In approximately 90% of EHEs, there is recurrent *WWTR1::CAMTA1* gene fusion resulting from t(1;3)(p36;q25). *YAP1::TFE3* gene fusion of EHE, a rare and morphologically distinct subtype, has been identified recently. This subtype is characterized by prominent vascular spaces, brightly eosinophilic cytoplasm, solid growth pattern, and a better prognosis (1, 2). Here, we describe an ultra-rare case of hepatic EHE with *TFE3* rearrangement and review the subset of EHE occurring at various sites in the literature, with the aim of better understanding of EHE and its unique subtype of *TFE3* rearrangement among clinicians, radiologists, and pathologists.

## 2 Case presentation

### 2.1 Materials and methods

#### 2.1.1 Clinical materials

**Figure 1:**
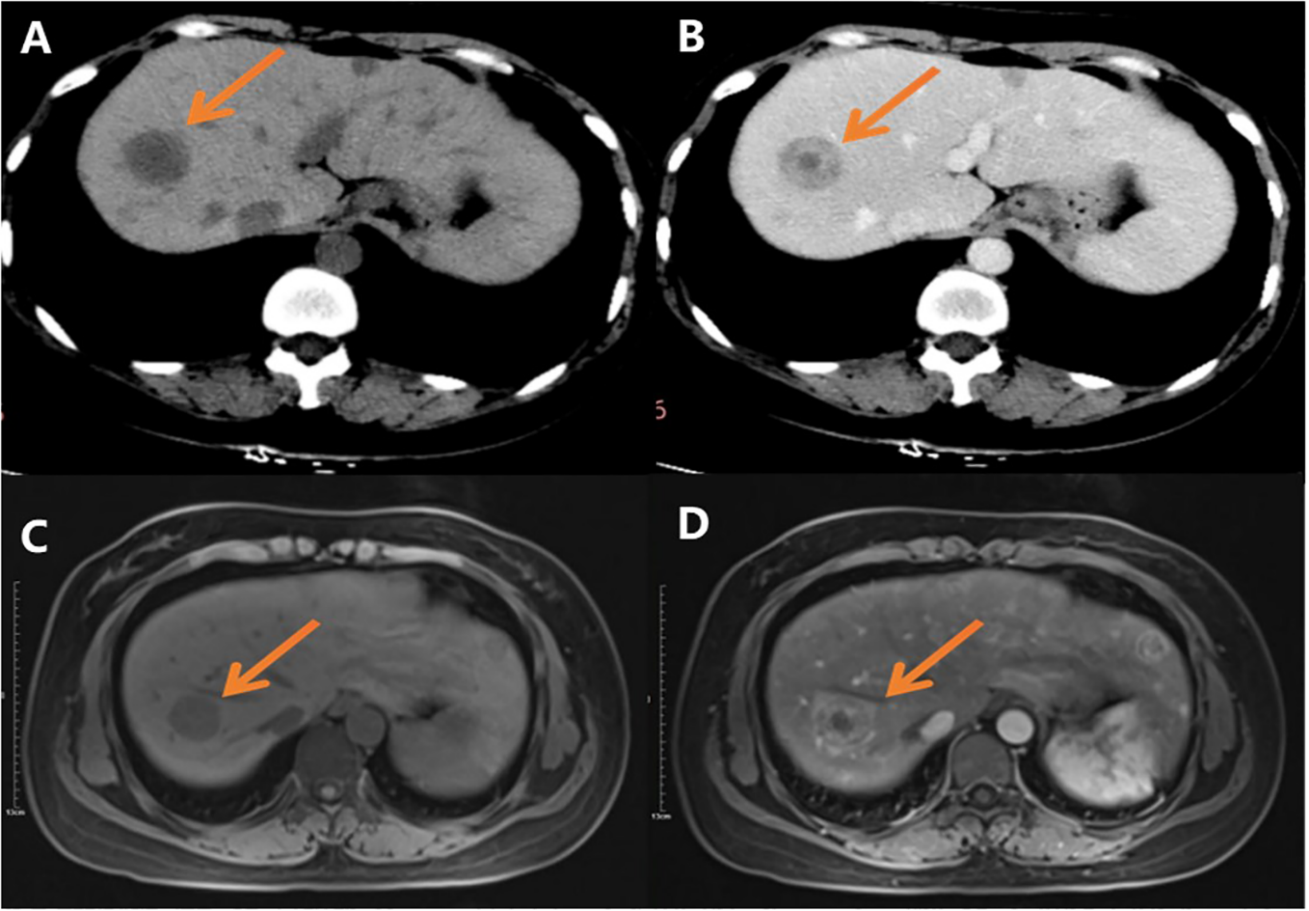
CT and MR images: **(A)** CT scan shows multiple round low-density shadows in the liver; **(B)** CT scan shows there is ring-shaped enhancement, and the “bull’s eye sign” can be seen after contrast administration; **(C)** Slightly low signal shadows in T1WI; **(D)** Slightly high signal shadows in T2WI.

A 36-year-old female presented with chest tightness two years ago, whose chest and abdominal CT revealed liver multiple occupations incidentally considered to be benign in an outside hospital. The patient did not receive any treatment. Subsequently, the patient was referred to our institution for further evaluation and management. A recent CT performed in our hospital showed multiple circular low-density shadows with ring-shaped enhancement (“bull’s-eye sign”) in the liver, the largest one was 28mm in diameter (**Figures 1A, B**). MRI showed multiple round-like low signals on T1WI and high signals on T2WI in the liver, with well-defined and ring-shaped enhancement (**Figures 1C, D**). Multiple metastatic tumors in the liver were diagnosed based on CT and MRI images. The patient underwent a CT-guided core needle biopsy.

#### 2.1.2 Methods

##### 2.1.2.1 Hematoxylin&eosin and immunohistochemistry

The biopsy specimens were routinely sampled, fixed in 10% neutral formalin, embedded in paraffin, sectioned, subjected to H&E and immunohistochemical staining, and finally microscopically observed. Immunohistochemical staining was performed by the EnVision method using antibodies to CD34, CD31, ERG, INI-1, FLi-1, AE1/AE3, Hepatocyte, CK7, CD117, DOG-1, and Ki-67. Negative and positive controls were set up for all experiments.

##### 2.1.2.2 FISH

*TFE3* gene rearrangement was detected by fluorescence *in situ* hybridization (FISH) using the LBP *TFE3* gene breakage probe, and the specific operation method was carried out regarding the instruction manual of the kit. The reagents for this test were purchased from Guangzhou Anbipin Medical Laboratory Company.

## 3 Results

### 3.1 Gross view

Two punctured tissues, 1.4 and 1.8cm in length separately, grayish red.

### 3.2 Microscopic view

**Figure 2:**
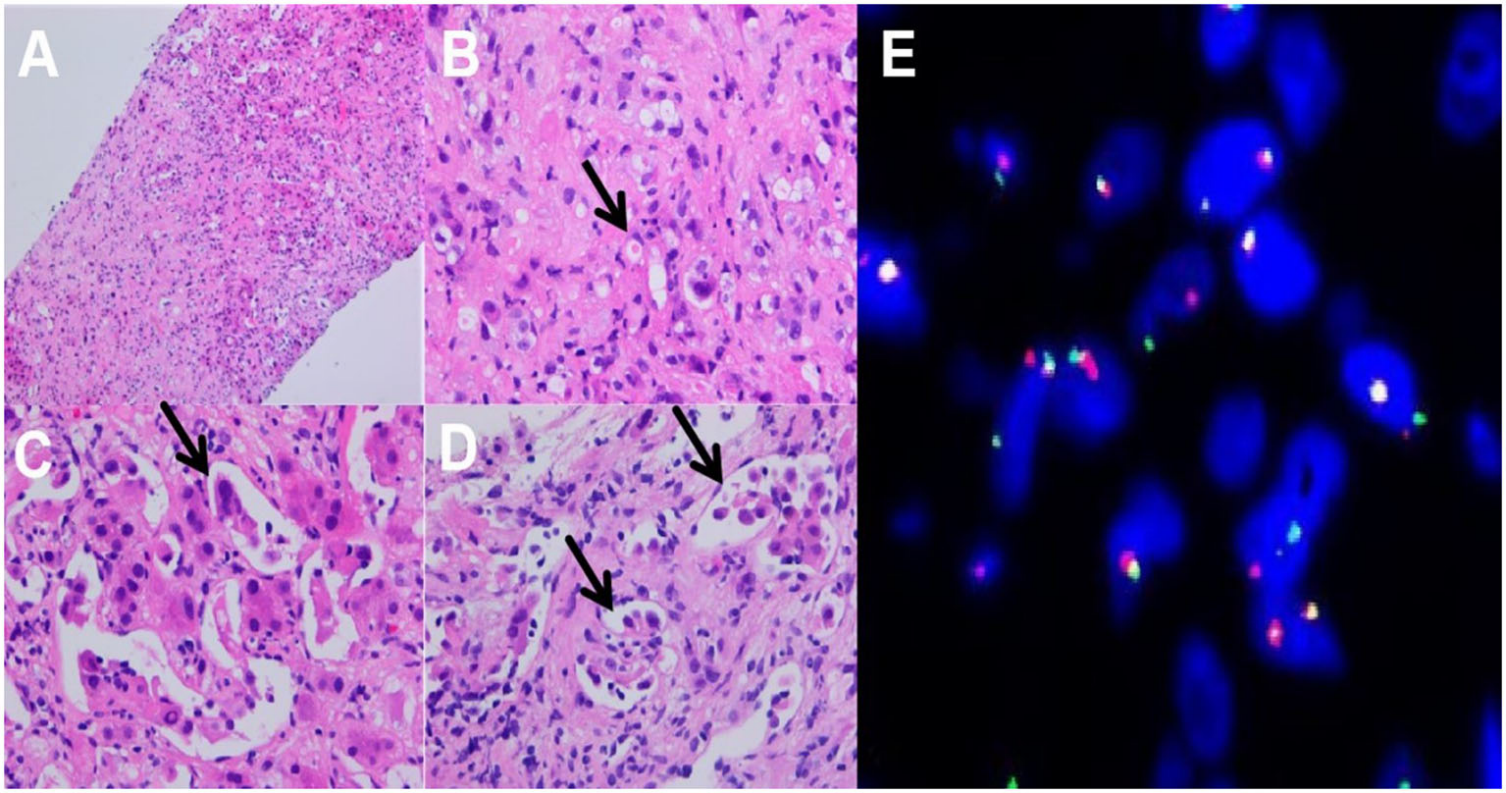
Histopathological features: **(A)** Focal fibrosis in liver tissue (HE-100X); **(B)** Tumor cells with cytoplasmic vacuoles or abundant eosinophilic cytoplasm, with small nucleoli, histiocyte-like, lipoblast-like, mildly heterogeneous, primitive vascular lumens, erythrocytes are seen in primitive lumens, single, striated, nested distributions, (HE-400X); **(C)** Tumor cells infiltrate to hepatic sinusoids (HE-400X); **(D)** high-power view showing arborizing structures lined with a tombstone or hobnailed tumor cells (HE-400X); **(E)** FISH testing:*TFE3* gene breakage rearrangement.

A Focal fibromyxoid matrix was observed in the liver tissue under low-power microscopy (**Figure 2A**). Epithelioid cells and plump spindle cells were seen in the myxohyaline stroma, hepatic sinusoids, and inner blood vessels. Lesional cells were arranged in scattered, nested, or cords. High-power microscopy showed tumor cells have moderate eosinophilic cytoplasm and round to plump spindle nuclei with inconspicuous nucleoli, histocyte-like, partially lipoblast-like cells with vacuolated cytoplasm (**Figure 2B**). Tumor cells infiltrated the hepatic sinusoids, with single vacuoles and erythrocytes in some cells (**Figure 2C**). A few tumor cells grew in distinct arborizing structure lined with tombstone or hobnailed tumor cells rather than typical well-formed vascular spaces. This morphology mimics the papillary intravascular lymphangioendothelioma (PILA) (**Figure 2D**). The tumor was accompanied by a small number of lymphocytic infiltrations, with no mitotic activity or necrosis in the core needle biopsy sample.

### 3.3 Immunophenotype

**Figure 3:**
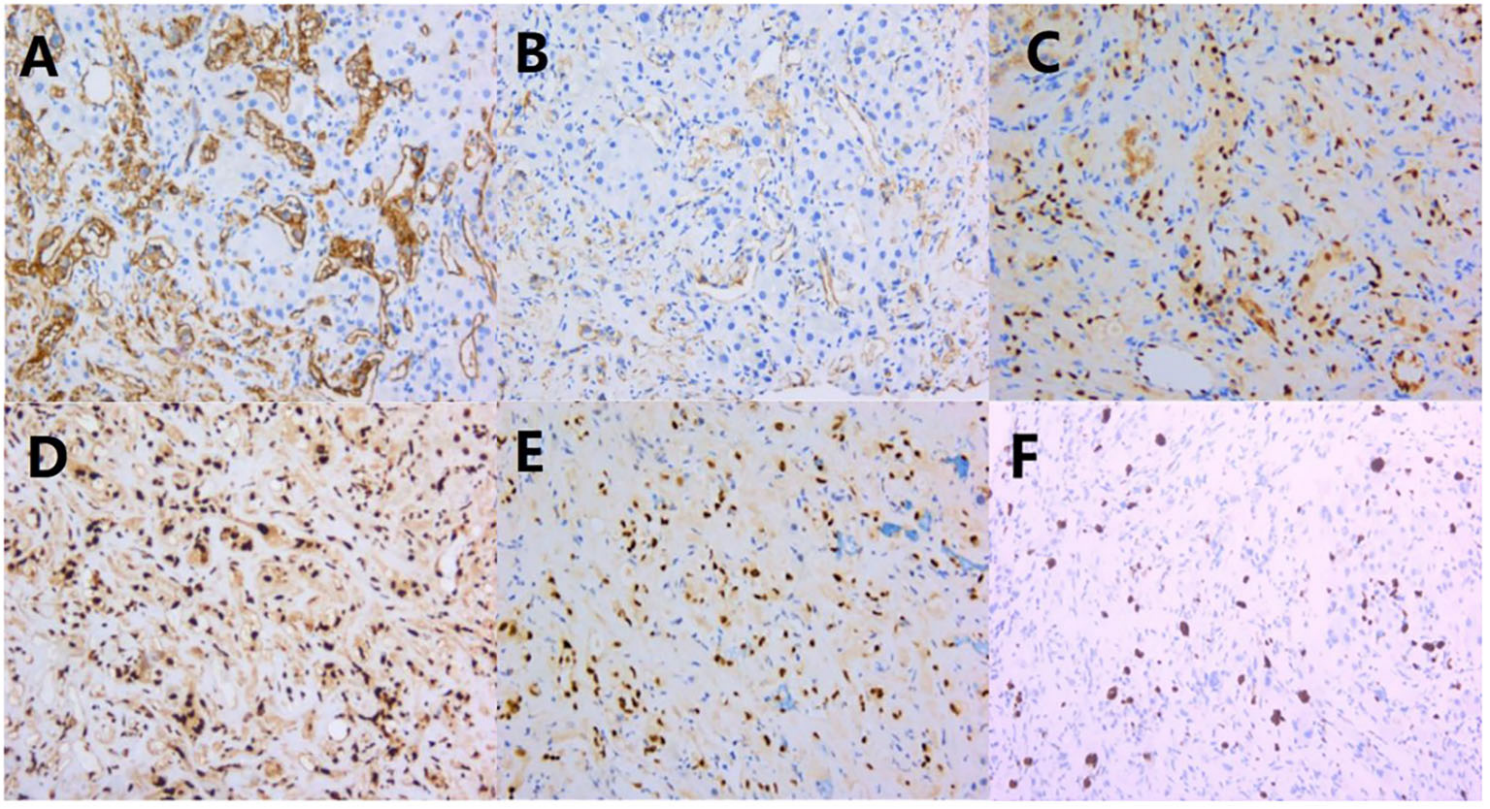
Immunohistochemical results: **(A–D)** CD34, CD31, ERG, and FLi-1 were diffusely and strongly positive; **(E)** TFE 3 was diffusely and strongly positive; **(F)** Ki-67 proliferation index was around 15%.

The tumor cells exhibited an endothelial cell phenotype with diffuse strong positivity for CD34, CD31, ERG, and FLi-1 (**Figures 3A–D**), and diffuse and strong nuclear reactivity to TFE3 (**Figure 3E**). INI-1 was retained. There was no expression of AE1/AE3(-), Hepatocyte (-), CK7(-), CD117(-), and DOG-1(-). Ki-67 label index was 15% (**Figure 3F**).

### 3.4 FISH testing

The results showed a broken rearrangement of the *TFE3* gene (**Figure 2E**).

### 3.5 Pathological diagnosis

Hepatic Epithelioid hemangioendothelioma with *TFE3* rearrangement.

## 4 Discussion

### 4.1 Clinical features

EHE of the liver is much rare, with an incidence of about 1/1 million (3, 4). The etiology and pathogenesis of hepatic EHE are still unclear. It may be related to alcohol consumption, oral contraceptives, Crohn’s disease, hepatitis, exposure to chemical substances such as polyvinyl chloride (5), monocyte chemotactic protein-1, and chronic Bartonella infection (6, 7).

Since *TFE3* rearrangements in EHE were first reported in 2013 by Antonescu et al. (8), they were found in various sites, such as soft tissue, bone, lung, liver, and inguinal lymph nodes (9–11). We summarized 50 cases of EHE with *TFE3* rearrangement in the literature including 3 hepatic ones. It occurred more frequently in young adults (mean age 39.5 years, range 14-65 years) with female predominance (**Table 1**). Its symptoms depend on the anatomical site of involvement and usually lack specificity. It may present as a painful mass in soft tissue. It is hard to recognize the primary malignant nature, frequently mistaken as benign or metastatic. For limited hepatic epithelioid hemangioendothelioma (HEHE) materials, patients do not have obvious symptoms, are often overlooked, and most of them are found incidentally during physical examination or routine US. A few patients may have abdominal pain, ascites, hepatomegaly, jaundice, emaciation, etc., with normal or slightly elevated blood AFP, CEA and tumor markers (CA199, CA125) (4, 21). Our patient was a young female who presented clinically with chest tightness, no abdominal distension or pain, and no significant change in weight. The lesions were located in the liver, presenting as multiple or metastatic, with normal blood AFP, CEA, CA199, and CA125. The clinical presentation is consistent with most of the literature.

**Table 1:** Clinical features associated with EHE with *TFE3* rearrangement.

Literature sources and cases in our group	Number of cases (n=50)	Sex (m/f)	Age (years)	Site of disease	Treatment	Prognosis
our group	1	Female	34	liver	No treatment, regular follow-up	No metastasis
Kuo FY (11)	1	Female	39	liver	Liver transplantation	No recurrence
Anderson WJ (1)	13	Female: 8 Male: 5	14-62	Limbs or trunk, bones, head and neck, lungs, etc.	NK	NK
Lotfalla MM (12)	1	Female	65	liver	Liver transplantation	NK
Zhang HZ (10)	2	Female	16/23	bones	Resection	No recurrence or metastasis
Rosenbaum E (9)	10	Female: 6 Male: 4	28-62	Soft tissue, lungs, bone, etc.	3 cases of resection; 2 cases of systemic treatment	6 cases of metastasis
Puls F (2)	1	Male	29	inguinal lymph nodes	Resection	No recurrence or metastasis
Flucke U (13)	2	Male	14/19	Inguinal lymph nodes/lungs	1 case of resection	NK
Lahori M (14)	2	NK	NK	NK	NK	NK
Thway K (15)	1	Female	40	Limbs, lungs, etc.	Chemotherapy+Resection	Recurrence
Shibayama T (16)	2	NK	NK	Bones, lungs, liver, etc.	NK	NK
Antonescu CR (8)	10	Female: 5 Male: 5	30 (median)	Soft tissue, lung, bone	NK	1 case of recurrence, 4 cases of metastasis
Song QY (17)	2	Female: 1 Male: 1	42/51	head and neck	Resection	1 case of recurrence
Abdelmogod A (18)	1	Male	40	Right calf extremities	Resection after metastasis	Metastasis
Liţescu M (19)	1	Female	37	mediastinum (anatomy)	Resection	No recurrence or metastasis
Patel NR (20)	1	NK	NK	NK		

NK, not known

### 4.2 Imaging findings

The imaging manifestations of the HEHE are diverse, ranging from single, multiple nodular to diffuse fusion lesions. Following the disease progression, multiple nodules can fuse into patches at a late stage, which may be related to hepatic vascular invasion. The US shows most of them are hypoechoic nodules with clear borders, hyperechoic nodules are seldom and imply hemangiomatous nodules. CT scans often reveal heterogeneous density nodules with low density center, forming the typical “halo sign”. Ring enhancement is seen in the arterial phase without central enhancement. On MRI, T1WI shows slightly hypointensity, and the central signal is even lower when the tumor is necrotic. T2WI shows hyperintensity with a higher central signal, and the tumor tissues are significantly enhanced. The enhancement modes of HEHE: ring enhancement, inhomogeneous enhancement, and flocculent enhancement. Among them, ring enhancement is usually the most common. At present, it’s believed that HEHE has multiple helpful imaging signs for the diagnosis. For example, black or white target sign, “pericardial crumpling sign”, early “intratumoral vascular sign”, and late “Lollipop sign” (22). The “pericardial crumpling sign” is important but not specific. The “Lollipop sign” was a characteristic manifestation of HEHE (23). Although we did not find the “pericardial crumpling sign” and “Lollipop sign” in our case, CT showed a “bull’s-eye sign” and MRI showed ring enhancement, which was consistent with literature reports.

HEHE needs to be differentiated from liver metastases, hepatocellular carcinoma (HCC), cholangiocellular carcinoma, and hepatic hemangioma before surgery. The enhancement manifestations of liver metastases are diverse, with circular enhancement being more common and easily confused with HEHE. Therefore, it is particularly important to closely combine the history of primary malignant tumors. Patients with HCC usually have a history of hepatitis B and/or liver cirrhosis, and the enhancement of the tumor is characterized by “fast-in-fast-out” enhancement without ring-shaped enhancement. AFP is commonly elevated in HCC, however, it is normal or mildly elevated in HEHE. In addition, most benign and malignant tumors of the liver rarely show the “lollipop sign”. Hepatic hemangioma has typical progressive cardiac enhancement characteristics, with homogeneous enhancement in the delayed phase, and usually does not show the “pericardial crumpling sign”. Although cholangiocellular carcinoma may present with “peritoneal crumpling sign”, features such as thickening of the bile duct wall, dilation of the bile duct lumen, and elevation of CA199 can be distinguished from HEHE. As the CT and MRI images of HEHE have certain imaging features, they help in the diagnosis and differential diagnosis of the lesions. In addition, PET-CT which is used to detect living tumor tissue and assess disease activity, is an essential imaging modality for tumor detection. However, probably due to the rarity of the disease, no PET-CT features of *TFE3*-rearranged EHE have been reported to date.

### 4.3 Histopathologic features

Histologically, EHE is a malignant tumor composed of vascular endothelial cells accompanied by a distinctive myxoid-hyaline transformation of the mesenchyme with *WWTR1::CAMTA1* and *YAP1::TFE3* fusion genes (8, 24, 25). Classic EHE often shows epithelioid or histiocyte-like cells arranged in a striated, nested, or single scattered pattern, with spindle, round, oval, or imprinted nuclei with fine chromatin, visible intracytoplasmic vacuoles containing erythrocytes. They generally do not form mature vascular lumens and are often characterized by *WWTR1::CAMTA1* fusion (13). The t(11; X)(q13;p11) translocation that occurs in rare cases forms the *YAP1::TFE3* fusion gene, and this subtype of EHE has several specific morphologic features, including the typical characteristics of solid or pseudo-glandular vesicle-like arrangement, well-formed blood vessels, and broad eosinophilic cytoplasm. The absence of well-formed vessels has also been reported in this rare type.

Nested, pseudo vesicular, partly solid growth pattern, occasional pseudo inclusion bodies, and interstitial inflammatory cell infiltration were observed in a single case (2, 8, 10). The core biopsy tissue in this case showed scattered, nest or cord arrangement of epithelioid to spindle cells, lipoblast-like or histiocyte-like cells, with eosinophilic cytoplasm with vacuolated cytoplasm. Our EHE with *TFE3* rearrangement is unique in the presence of clear cells, lipoblast-like cells and focal PILA-like morphology, but without the characteristic well-defined vessels. The PILA-like morphology first reported by song et al. is ultra rare in *TFE3*-rearranged EHE (17). The PILA-like tumor cells express *TFE3* to the same extent as other tumor cells. We also consider it as PILA-like morphology rather PILA.

Immunoreactivity for keratins (CK7 and CK18) is observed in the majority of EHEs (50% and 100%, respectively), while CK8 was seen in 10% cases and CK14 and CK19 in none. Epithelial membrane antigen (EMA) was occasionally detectable in EHE (26). TFE3 immunoreactivity was positive but lack of specific, which can also be positive in *WWTR1::CAMTA1* EHE. *TFE3* rearrangement requires FISH assay confirmation (13). Positive expression of endothelial cell markers such as CD31, CD34, ERG, and FLI-1 are commonly detected by immunohistochemistry. CD34 has high sensitivity but poor specificity. The positive rates of CD34 and CD31 are 94% and 86%, respectively (27). CD34 and CD31 should be used as the first choice for diagnosis. Ki-67 has a low proliferation index. FISH assay showed that the *TFE3* gene was broken and rearranged. We diagnose this case as EHE with *TFE3* rearrangement based on immunophenotype and the FISH result. Regarding prognosis, characteristics such as nuclear pleomorphism, mitotic activity, necrosis, and Ki-67 index greater than 10-15% were correlated with aggressive behavior for EHE. Rosenbaum E et al. proposed the two-grade histologic system of classical EHE based on nuclear pleomorphism, mitotic activity, and necrosis in *WWTR1::CAMTA1* fused EHE (9). There is no distinctive histologic grading of EHE with *TFE3* rearrangement. Our patient had mild nuclear pleomorphism, a lack of mitotic figure, and no necrosis. It may be grade 1, If we apply the classic EHE grading system.

### 4.4 Differential diagnosis

*TFE3*-rearranged EHE often need to be differentiated from other similar tumors: (1) Epithelioid angiosarcoma (EA) is a malignant tumor of vascular endothelial origin. Immunohistochemistry is not helpful in differentiating between the two tumors. EA is common in older men instead of young women in EHE. Both of them can appear as epithelioid cells with solid growths. EA is much more malignant, with more striking atypical. MYC amplification occurs in a subset of EA. *YAP1::TFE3* fusions is suggestive of EHE. (2) Epithelioid sarcoma (ES) is a malignant mesenchymal tumor with predominantly epithelioid, spindle-shaped, and rhabdomyosarcoma-like cells. The mitotic figure is often <5/10HPFs. ES show immunoreactivity for keratin and EMA, especially CK8 and CK19. Diffuse loss of SMARCB1(INI-1) expression is specificity. FLi-1, ERG, and CD34, nonspecific markers of vascular differentiation, could be expressed in more than half of ESs. In contrast, ES is negative for CD31 (28). *SMARCB1* deletion detected by FISH or NGS is characteristic. (3) Pseudomyogenic hemangioendothelioma (PMHE) is similar to *TFE*3-rearranged EHE in morphology, with epithelioid to plump spindle-shaped cells, eosinophilic cytoplasm, and inflammatory cell infiltration in the stroma. Immunohistochemically they both express ERG and Fli-1, differing in that PMHE expresses the highly sensitive marker FOSB and does not express CD34. The FISH assay revealed a rearrangement of the *FOSB* gene, and its NGS showed a *SERPINE1::FOSB* gene fusion with some specificity (29). (4) Adenocarcinoma, especially intrahepatic cholangiocarcinoma (iCCA) in the liver, may appear as multifocal lesions on imaging. Diagnosis is quite challenging in small core biopsy, especially when the nuclei atypia is mild. Immunohistochemistry staining is essential to distinguish them. iCCA expresses biliary lineage-defining markers (CK7 and CK19). EHE can be easily misdiagnosed as iCCA especially in puncture samples. They both express CK7, while EHE shows negative for CK19. It is important to do a panel of CK7 and CK19 as well as vascular markers in atypical cases to determine the diagnosis of iCCA. Notably, it is important to rationally interpret *TFE3 r*earrangements in the context of morphology, as *TFE3* rearrangements are also seen in other tumors such as alveolar soft tissue sarcomas, Xp11.2 translocation-associated renal cell carcinomas, and perivascular epithelioid cell tumors.

### 4.5 Treatment

Half of the untreated EHE patients had stable disease when the lesions first appeared in the liver or lungs. For single lesions, especially in bone or liver, 75% of patients showed no evidence of recurrence after timely surgical resection. Systemic therapy is necessary for symptomatic multifocal, metastatic, observation-failed or postoperative recurrent patients. Antiangiogenesis agents, cytotoxic agents, and anti-PD-1 antibody are recommended (9). EHE patients with *TFE3* rearrangements are generally younger and have a much better prognosis than the classical *WWTR1::CAMTA1* type, with longer survival but a higher risk of metastasis (9, 19). *TFE3* rearranged EHE is still an aggressive neoplasm with a 6% (3/50) local recurrence rate and a 22% (11/50) metastasis rate, even after the surgery (**Table 1**). Multifocal hepatic EHE with WWTR1::CAMTA1 is monoclonal representing metastatic implants of the same neoplastic clone rather than a synchronous occurrence of multiple neoplastic clones (30). However, whether multifocal TFE3 rearranged EHE of liver represents a pattern of metastasis or multiple separate primary tumors remains to be elucidated. Due to its rarity, more cases should be collected for further research in multicenter. There are only two cases of hepatic EHE with *TFE3* rearrangement reported in the English literature. Both presented multiple liver masses and underwent liver transplants. Postoperative pathology confirmed EHE. One patient showed no signs of recurrence or metastasis during the 13 years of follow-up after liver transplants (11). Our patient denied any treatment and received regular CT examination. After 3.5 years of follow-up, the lesion stabilized (**Table 2**). Further research is warranted to determine if the *TFE3* rearrangement predicts a less aggressive course than the *WWTR1::CAMTA1* fusion.

**Table 2:** Patient treatment process.

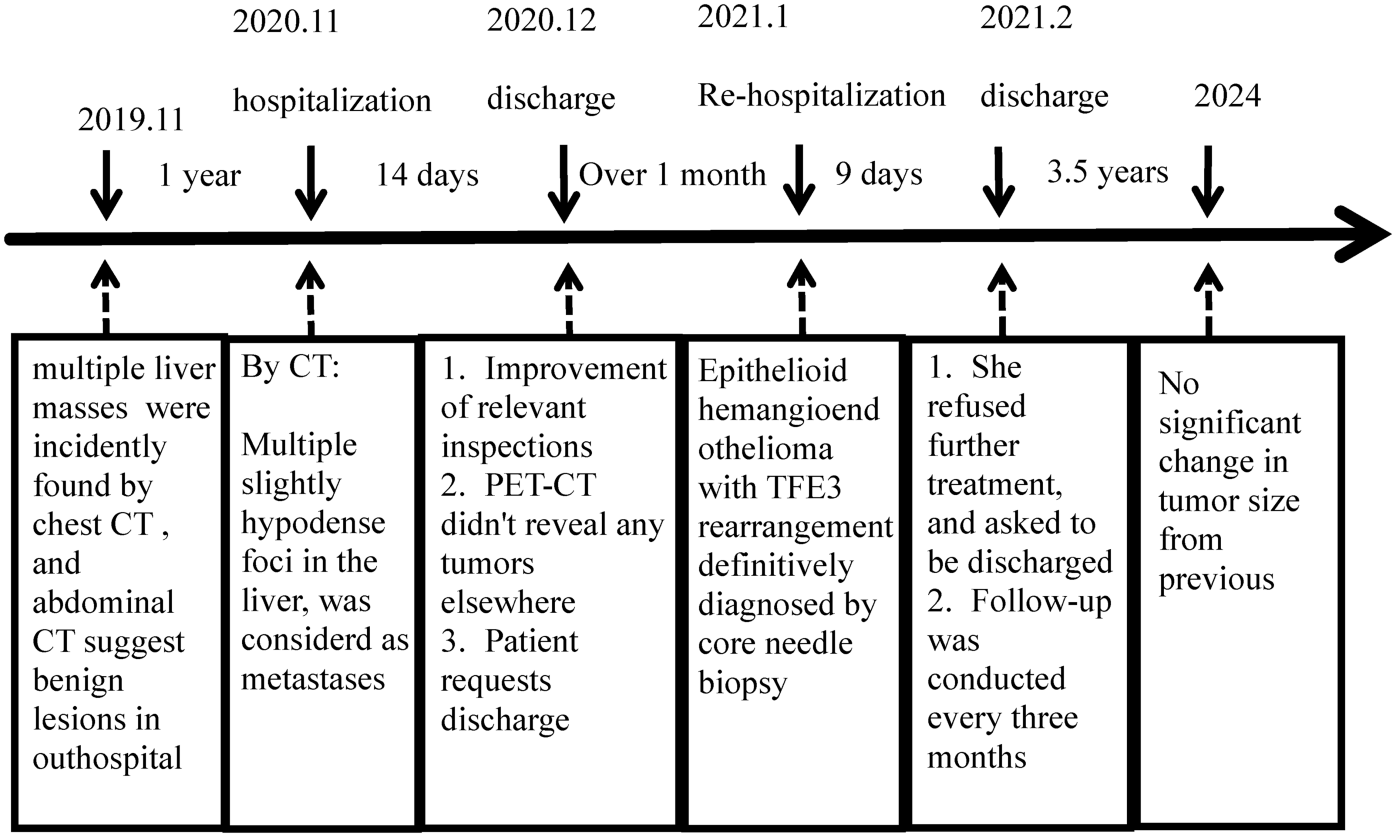

*TFE3* rearrangements are characteristic of alveolar soft part sarcomas (ASPS), Xp11.2 translocation renal cell carcinomas (Xp11-RCC), besides EHE with *TFE3* rearrangement. *TFE3* immunohistochemistry lacks specificity and sensitivity in the diagnosis of *TFE3* rearrangement. Final diagnosis needs fluorescent *in-situ* hybridization (FISH) and other molecular tests (31). Although the fusion partner of *TFE3* is unknown in this case, previous studies of other tumors have shown that *TFE3* always fused to the house-keeping gene. *TFE3* contributes its DNA-binding domain, with imputed function in the fusion. House-keeping’s promoter leads *TFE3* misexpression to promote tumorigenesis (32). However, *TFE3* is not readily targetable. The mammalian target of rapamycin (mTOR) as *TFE3* upstream regulator is targetable in some *TFE3* rearranged tumor, like Xp11-RCC and ASPS. mTOR inhibitors (sirolimus) maybe an efficacious systemic treatment for EHE (33, 34). Correct diagnosis is essential for treatment. Unlike the less malignant EHE, hepatic angiosarcoma is a highly aggressive vascular tumor for which surgery is the most effective treatment. However, radical resection is difficult for multifocal lesions. Chemotherapy, tyrosine protein kinase inhibitors (TKI) therapy and immunotherapy may be effective.

There are some shortcomings in our study. First, although *TFE3* rearrangement was confirmed by the FISH technique. Next-generation sequencing and RT-PCR were not performed to detect and validate *TFE3*’s partner gene. We can’t further study the oncogenic mechanism underlying the fusion of *TFE3* and its partner and likely involved signal pathway. Second, we were unable to obtain samples from different liver nodules to verify whether multifocal lesions were monoclonal or polyclonal, and failed to figure out if it was metastasis or multiple primary tumors.

In conclusion, Hepatic multiple lesions with ring-enhancement should be considered as a possibility for EHE, besides metastases and primary liver cancer in imaging. Histologically,*TFE3*-rearranged EHE has distinct morphology features. PILA-like morphology is rarely described. Absence of well-formed vessels adds the difficult to diagnosis as well, especially in core biopsy. *TFE3* immunostaining can be used as a screening tool, but is not specific for *TFE3-*rearranged EHE, and the final diagnosis is dependent on molecular testing. At present, both hepatic EHE with *TFE3* rearrangement present multifocal lesions, and it is unclear whether they are metastatic or multiple primary tumors. More cases should be collected to verify this issue. Further study on the oncogenic mechanism underlying the fusion of *TFE3* and its partner and the likely involved signal pathway is warranted to uncover its pathogenesis and target therapy. We recommend every 3 to 6 months follow-up for 2 to 3 years by CT and/or MRI with contrast according to National Comprehensive Cancer Network guidelines. Systemic treatment is recommended in the presence of multiple lesions or symptoms. Antiangiogenesis agents, cytotoxic agents, and anti-PD-1 antibody are recommended. mTOR inhibitors (sirolimus) maybe an efficacious systemic treatment for EHE as well. If acceptable, liver transplantation may be curative.

## Data Availability

The original contributions presented in the study are included in the article/supplementary material. Further inquiries can be directed to the corresponding author.
